# Clinical and radiological analysis of a series of periapical cysts and periapical granulomas diagnosed in a Brazilian population

**DOI:** 10.4317/jced.53196

**Published:** 2017-01-01

**Authors:** Daniel-Petitet Tavares, Janderson-Teixeira Rodrigues, Teresa-Cristina-Ribeiro-Bartholomeu dos Santos, Luciana Armada, Fábio-Ramôa Pires

**Affiliations:** 1 DDS, Post graduation Program in Dentistry, Estácio de Sá University; 2 DDS, MSc, Post graduation Program in Dentistry, Estácio de Sá University; 3 DDS, MSc, Professor, Oral Pathology, State University of Rio de Janeiro; 4 DDS, MSc, PhD, Professor, Post graduation Program in Dentistry, Estácio de Sá University

## Abstract

**Background:**

Periapical cysts (PC) and periapical granulomas (PG) are the two most common chronic inflammatory periapical diseases, but their clinicoradiological characteristics can vary depending on the methods employed in each study. The aim of the present work was to analyze the clinical and radiological profile of a series of PC and PG diagnosed in a Brazilian population.

**Material and Methods:**

The files of two Oral Pathology laboratories were reviewed and all cases diagnosed as PG and PC were selected for the study. Clinical and radiological information were retrieved and data were tabulated and descriptively and comparatively analyzed.

**Results:**

Final sample was composed by 647 inflammatory periapical lesions, including 244 PG (38%) and 403 PC (62%). The number of women affected by PG was significantly higher than the number of women affected by PC (*p*=0.037). Anterior region of the maxilla was the most common affected area for both entities (39% of the cases), but the most common anatomical location of PG (anterior maxilla and posterior maxilla) was different from PC (anterior maxilla and posterior mandible) (*p*<0.0001). Upper lateral incisor was the most affected tooth. The mean radiological size of the PC was larger than the mean radiological size of the PG (*p*<0.0001) and PC showed well-defined radiological images more frequently than PG (*p*<0.0001).

**Conclusions:**

PC were more common than PG, both showed predilection for adult females, most lesions affected predominantly the anterior maxilla and PC presented larger mean radiological diameter and well-defined images when compared with PG.

** Key words:**Periapical granuloma, periapical cyst, radicular cyst, diagnosis, Oral Pathology.

## Introduction

Periapical granulomas (PG) and periapical cysts (PC) are considered the most common periapical intraosseous chronic diseases. Their origin is associated with pulp infection and necrosis due to the presence of microrganisms and progression of the infectious and inflammatory process to the periapical region. Several studies have been performed in order to detail the clinical, anatomical and radiological characteristics of PG and PC in large series, but results show a wide variation due to specific loco-regional featu-res and methodological limitations from each study (1-10).

Few informations are available about the frequency and epidemiological distribution of PG and PC in Brazilian populations and their similarities and differences comparing with other populations. Thus, the aim of the present study was to descriptively and comparatively analyze the clinical and radiological features from a series of PG and PC diagnosed in two Oral Pathology laboratories in Rio de Janeiro, Brazil.

## Material and Methods

The files of the Oral Pathology laboratories, Estácio de Sá University and State University of Rio de Janeiro, were reviewed, respectively, from 1998 to 2013 and from 2005 to 2013, and all cases diagnosed as PG and PC were selected. All cases were obtained through surgical procedures (periapical surgery or extraction) and the specimens were immersed on 10% buffered formalin for laboratorial processing, including basic routine steps and final analysis of 5 µm hematoxylin and eosin stained sections on histological slides through light microscopy. All cases were histologically reviewed to confirm diagnosis and only the cases presenting histological, clinical and radiological features allowing final diagnosis as PG or PC were included. Histologically, PG were characterized by the presence of a fibrous connective tissue containing a granulation tissue with small blood vessels, fibroblasts and chronic inflammatory cells, mostly lymphocytes, plasma cells and macrophages. PC showed similar features than PG associated with the presence of a complete or partial cystic cavity lined by a non-keratinizing squamous stratified epithelium of variable thickness showing exocytosis (11). In some cases, apart from the above described features, both PG and PC showed a mixed inflammatory infiltrate, with a prominent neutrophilic infiltrate. Exclusion criteria were the existence of tissue samples insufficient for final correct diagnosis, highly fragmented specimens difficulting analysis of the epithelial lining, laboratory forms containing no clinical and radiological information and cases in which final diagnosis changed to other conditions apart from PG and PC after histological review.

All laboratory forms from the selected cases were reviewed and information about gender, age, anatomical location of the lesions (anterior or posterior mandible, anterior or posterior maxilla), teeth associated with the lesions, time of complaint, local swelling, symptoms (pain and/or purulent discharge), radiological limits of the lesions (well or ill defined, according with the presence of a border of bone sclerosis), largest radiological diameter of the image (in milimeters) and final diagnosis (PG or PC).

Data were descriptively and comparatively analyzed through the use of the software Statistical Program for Social Sciences (SPSS, version 17, Chicago, Illinois, United States), considering 5% (*p*<0.05) as the significance level and using chi-square and T test. The study protocol was submitted to and approved by the Ethics in Research Committee from the State University of Rio de Janeiro and approved under the number 536.544.

## Results

Final sample was composed by 647 inflammatory periapical lesions, including 244 PG (38%) and 403 PC (62%). Females represented 56% of the sample, and 61% and 53% of the patients affected by periapical granulomas and periapical cysts, respectively (*p*=0.037). Mean age of the patients was 41.5 years, ranging from 5 to 90 years.

Anatomical distribution of the lesions showed that 39% of them affected the anterior maxilla, followed by the posterior mandible (28%) and posterior maxilla (23%). PG affected mostly the anterior maxilla (34%) followed by the posterior maxilla (32%) while PC were diagnosed mainly in the anterior maxilla (43%) and posterior mandible (28%) (*p*<0.0001) ( Table 1). Anatomical location of PG and PC did not show statistical significant difference when comparing males and females (*p*=0.210). The upper right lateral incisor (60 cases, 11%) was the most affected tooth, followed by the upper left lateral incisor (56 cases, 10%), lower right first molar (46 cases, 8%), upper right central incisor (40 cases, 7%), lower left first molar (35 cases, 6%) and upper left central incisor (32 cases, 6%).

**Table 1:**
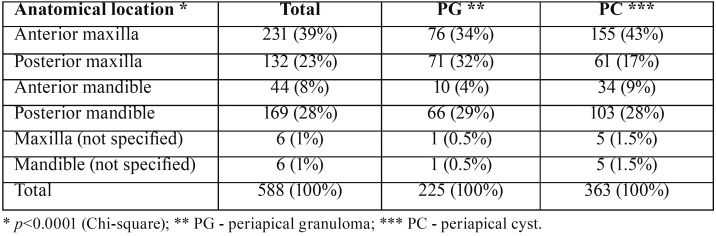
Anatomical distribution of the periapical granulomas and cysts.

Time of complaint was reported in 77 cases and showed a mean of 31.6 months (ranging from 1 to 240 months) with no statistically significant difference on the mean time of complaint of PG (19 cases, 37.9 months) and PC (58 cases, 29.6 months) (*p*=0.460). The presence of symptoms was reported in 207 cases and there was no statistically significant difference when comparing the number of asymptomatic and symptomatic PG and PC (*p*=0.287). Local swelling was reported in 132 cases and there was no statistically significant difference when comparing PG and PC presenting local swelling or no (*p*=0.468).

A total of 394 cases presented adequate radiographs for analysis and great diameter of the lesions ranged from 1 to 70 mm (mean of 13 mm). Mean size of PG (n=158) was 8.24 mm, in comparison with mean size of 16.24 mm for PC (n=236) (*p*<0.0001). Radiological limits of the lesions were evaluated in 200 cases and 148 (74%) and 52 cases (26%) showed, respectively, well and ill defined images. PG (n=41) were considered well defined in 25 cases (61%), while PC (n=159) were considered well defined in 123 cases (77%) (*p*=0.033). Gross information of the submitted specimens showed that their mean size ranged from 4 and 21.000 mm3 (mean of 891 mm3) and the mean size of PG (316.12 mm3) was lower than PC (1260.11 mm3) (*p*=0.0001). Presence of the endodontic treatment was observed in 206 cases.

Comparative analysis of the mean age of the patients by gender, presence of symptoms, presence of local swelling, radiological limits of the lesions and final diagnosis showed that there was a statistically significant difference only when comparing patients presenting lesions with well or ill defined radiological limits ( Table 2).

**Table 2:**
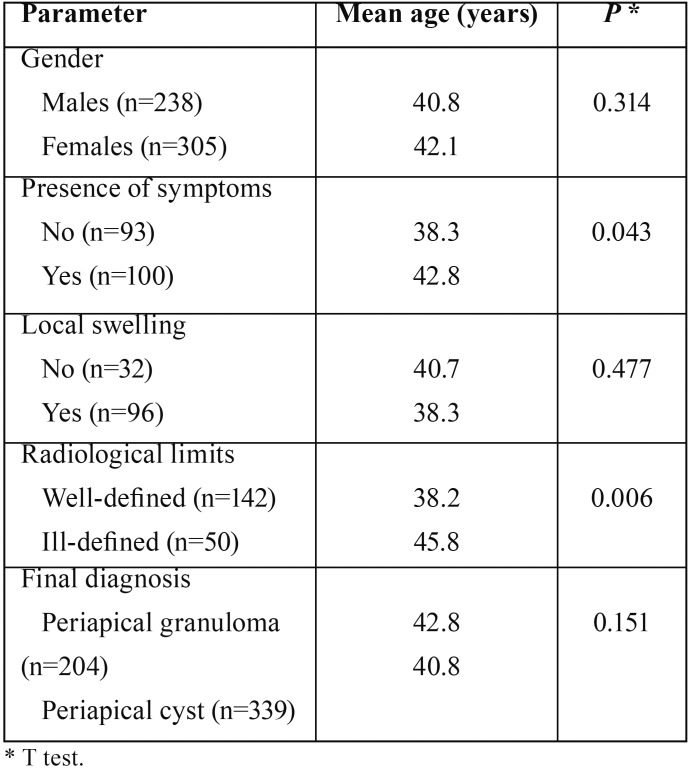
Mean age of the patients affected by periapical granulomas and cysts according with gender, presence of symptoms, presence of local swelling radiological limits and final diagnosis.

## Discussion

PC are inflammatory odontogenic cysts that represent from 35 to 87% of all odontogenic cysts, when analyzing studies derived from different worldwide populations ( Table 3). Previous studies showed that PC have not gender predilection or a slight male preference, most affected patients are in the third to fifth decades of life and the maxilla, especially the anterior region, is the site of predilection for the lesions (12-26). Studies derived from Brazilian populations have shown similar results (27-29).

**Table 3:**
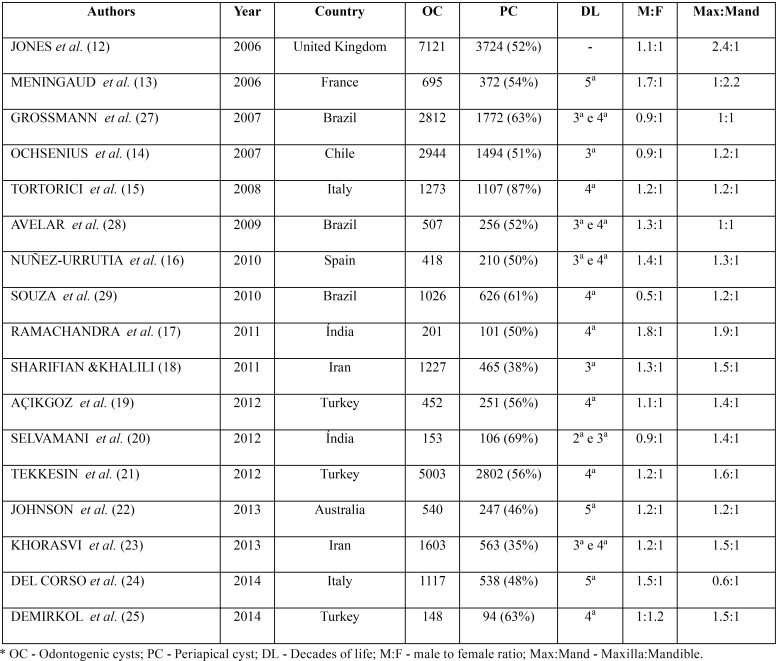
Frequency and age, gender and anatomical distribution of inflammatory periapical cysts reported in the literature from 2006 to 2014.

Several studies have focused the clinico-radiological profile of inflammatory periapical lesions, but results can be conflictant and show a wide range of variation, due to the different methods used and particularities from the studied population. The only accepted criteria for reliable differentiation of PG and PC is histological analysis, so all studies used for comparison are based on samples submitted to microscopical evaluation (7,11,21). PG are considered the most common inflammatory periapical lesions, representing up to 77% of the samples, and PC represent from 15 to 46% of the cases ( Table 4) (1-10). In addition, Stockdale & Chandler (2) reported a review of the published papers from 1954 and 1980 and showed that the frequencies of PC ranged from 7 to 54% and the frequencies of PG ranged from 45 to 93% of the cases. All these previously published samples do not reflect the true incidence of PC and PG in each population, as the results are based on the inflammatory periapical diseases that were surgically managed and submitted for histological evaluation (lesions associated with extracted teeth or derived from periapical surgery), leaving selection biases, and did not include the cases that responded favorably to conventional non-surgical endodontic therapy alone. However, studies based on these methods are the only source for reliable differentiation between PG and PC (8).

**Table 4:**
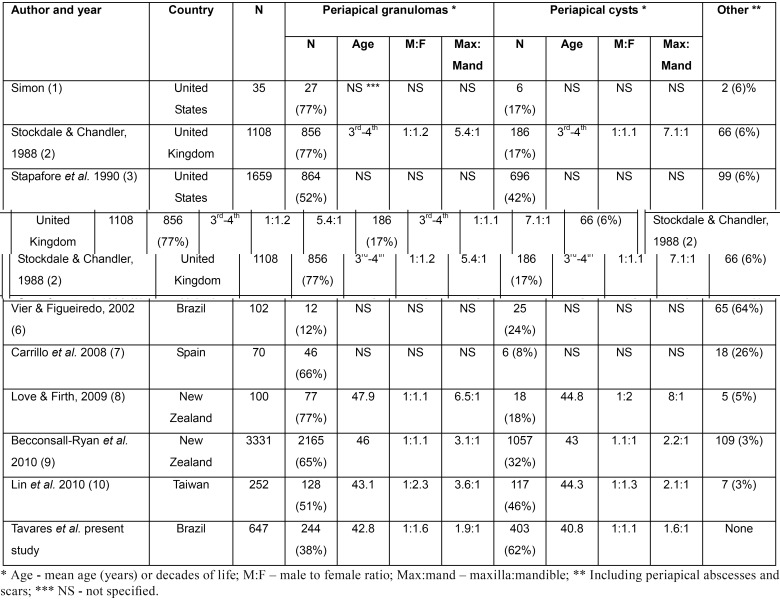
Frequency and gender, age and anatomical distribution of inflammatory periapical diseases reported in the literature.

Several methodological reasons can be listed to explain the diversity of the published results and the difficulties in comparing results from different sources. Simon (1) and Nair *et al.* (5) studied the frequency of inflammatory periapical diseases adhered to the roots of extracted teeth, not including lesions derived from periapical surgery of surgical procedures in which the tooth is submitted separated from the lesion. Besides that, this method is capable of classifying the removed cysts in “true” (cyst cavity without direct contact with the tooth apex) or “bay/pocket (cyst cavity in direct contact with the tooth apex) cysts. Nobuhara & Del Rio (4) included only inflammatory periapical lesions associated with teeth with failure in the conventional endodontic, while Vier & Figueiredo (6) included only inflammatory periapical lesions associated with teeth without endodontic treatment. Love & Firth (8) have shown that the frequency of PC did not depend on the condition of the associated teeth (primary or secondary canal infection) in teeth with persistent inflammatory periapical disease.

The results of the present study showed that PC were more common than PG, but this can be at least partially explained by the fact that some small inflammatory periapical lesions can be lost/deteriorated during laboratory processing or not submitted to laboratorial analysis by the clinicians/oral surgeons/endodontists responsible for the surgical procedure. This bias can reduce the number of small inflammatory periapical lesions diagnosed and, consequently, favoring a relative increasing of the number of PC. Other important aspect is associated with the expected more favorable biological response of smaller periapical lesions to conventional non-surgical endodontic treatment. Although there is no definitive biological evidence that PG respond better to conservative non-surgical management when comparing with PC, it is supposed that, as the former preceeds the latter, the possibility of favorable biological response is greater with PG. Thus, inflammatory periapical lesions submitted to surgical management, and consequent histological analysis, are preferentially the ones refractory to conventional endodontic treatment, including PC more commonly than PG.

Stockdale & Chandler (2) demonstrated that when the clinico-radiological hypothesis was PC, histological diagnosis was correct in 41% of the cases, while when the hypotheses was PG this value was 81%. Becconsall-Ryan *et al.* (9) showed that for the cases with a clinico-radiological provisional diagnosis of PG or PC, histological diagnosis confirmed the clinical suspicion in 72% and 50% of the cases, respectively, reinforcing that importance of submitting all specimens derived from periapical surgery to histological analysis.

Another methodological bias that could influence the results from different studies is the criteria used for histological classification of the inflammatory periapical lesions. Some studies have classified PG with acute inflammatory infiltrate as periapical abscesses and not as PG, rising the number of periapical abscesses (5,6). In the present sample, PG and PC that fulfilled the necessary histological criteria, even when associated with the presence of an acute inflammatory infiltrate suggestive of abscess formation, were classified as PC or PG. Additionally, Nobuhara & Del Rio (4) considered as PG the cases showing a connective tissue with peripheral proliferation of epithelium with no clear identification of a cyst cavity. Some of the images included on this particular study showed histological sections that would be possibly classified as PC by many oral pathologists, reinforcing the difficulties in establishing the proper histological criteria for this group of lesions.

One of the limitations of the studies evaluating the frequency of histologically diagnosed PG and PC is that some PG could in fact be PC that were correctly evaluated due to inadequate splicing or PC with small cavities that were not included on the sectioning axis. Serial sectioning is advisable to reduce these biases but, although they can be used in experimental studies, they shoud be used with caution in specimens derived from routine laboratorial diagnosis due to ethical and legal issues (5). In addition, most of the samples sent for routine histological analysis are composed by multiple fragmented specimens derived from surgical curettage, difficulting the identification of the epithelial-lining cystic cavity.

Although females were most affected by both PG and PC in the present sample, in accordance with the literature, these entities usually do not show any marked gender predilection (2,4,7,8,10). Mean age of the patients affected by PG and PC are in the fourth to fifth decades of life, in accordance with the results of the present study (2,4,7-10). The prevalence of these entities for patients in these age groups is probably associated with the progression of the infections on the root canal system, inadequate endodontic treatment/retreatment and need of extractions due to decays and periodontal disesase in teeth with concomitant endodontic involvement.

The anterior maxilla was the most affected region by both PG and PC, similarly to the data retrieved from the literature (2-4,8-10). Meningaud *et al.* (13) and Del Corso *et al.* (24) reported that PC predominantly affected the mandible, in contrast with most published studies. Our results showed that the distribution of the anatomical location comparing PG and PC was significantly different. The reasons that lead to this distinct anatomical distribution are unknown, but one could speculate that PC are more common in the posterior mandible because clinicians and endodontists indicate extractions more frequently than endodontic treatment to molars with large periapical lesions due to technical difficulties. The most affected teeth were the upper lateral incisors, the upper central incisors and the lower first molars, in accordance with the literature (2,10,11). The predilection for upper lateral incisors can be justified by the high frequency of caries, fillings and dentoalveolar trauma on these teeth, apart from the presence of a wide range of anatomical variations (apical curves, accessory channels and apical deltas), difficulting complete microrganisms removal and adequate obturation of the pulp chamber (2). The presence of signs and symptoms associated with PG and PC, such as pain, local swelling and purulent discharge, showed no statistically significant differences when comparing PG and PC from the present sample.

It has been accepted that most inflammatory periapical diseases respond to conventional non-surgical endodontic therapy and, as the exact pathogenesis of PG and PC is still unknown, the mechanisms that modulate their formation are incompletely described (30). The most accepted theory considers that the epithelial odontogenic remnants on the periodontal ligament (epithelial rests of Malassez) proliferate in response to the inflammatory stimuli derived from the persistent intraradicular infection. Cytokines and growth factors secreted by the components of the granulation tissue that characterizes the PG stimulate the epithelial proliferation up to the formation of central areas of degeneration on the proliferative epithelial islands, leading to the development of the cystic cavity (30). Therefore, it has been accepted that PC are originated from PG, but it is not known which PG will progress to PC. Both PG and PC are radiologically characterized by well defined unilocular radiolucent areas associated with the periradicular region from a non-vital tooth (10). Due to the previously cited accepted theory for development of PC, they tend to be larger and to show a peripheral area of bone sclerosis more frequently than PG (6,7), both features in accordance with the present results.

The results from the present study showed that PC were more common than PG and that both entities were more common in females on their fourth to fifth decades of life. The number of females affected by PG was higher than affected by PC and the anterior maxilla was the most common affected site by both PG and PC, with the upper lateral incisor as the most affected tooth. PC were radiologically larger in comparison to PG and showed well-defined limits more frequently than the latter.
